# Texture Evolution in AA6082-T6 BFSW Welds: Optical Microscopy and EBSD Characterisation

**DOI:** 10.3390/ma12193215

**Published:** 2019-10-01

**Authors:** Abbas Tamadon, Dirk J. Pons, Don Clucas, Kamil Sued

**Affiliations:** 1 Department of Mechanical Engineering, University of Canterbury, Christchurch 8140, New Zealand; dirk.pons@canterbury.ac.nz (D.J.P.); don.clucas@canterbury.ac.nz (D.C.); 2 Fakulti Kejuruteraan Pembuatan, Universiti Teknikal Malaysia Melaka, Durian Tunggal 76100, Malaysia; kamil@utem.edu.my

**Keywords:** AA6082-T6, bobbin friction stir welding, microstructure, optical microscopy, EBSD

## Abstract

One of the difficulties with bobbin friction stir welding (BFSW) has been the visualisation of microstructure, particularly grain boundaries, and this is especially problematic for materials with fine grain structure, such as AA6082-T6 aluminium as here. Welds of this material were examined using optical microscopy (OM) and electron backscatter diffraction (EBSD). Results show that the grain structures that form depend on a complex set of factors. The motion of the pin and shoulder features transports material around the weld, which induces shear. The shear deformation around the pin is non-uniform with a thermal and strain gradient across the weld, and hence the dynamic recrystallisation (DRX) processes are also variable, giving a range of observed polycrystalline and grain boundary structures. Partial DRX was observed at both hourglass boundaries, and full DRX at mid-stirring zone. The grain boundary mapping showed the formation of low-angle grain boundaries (LAGBs) at regions of high shear as a consequence of thermomechanical nature of the process.

## 1. Introduction

### 1.1. Context

Friction stir welding (FSW) [1,2] is a solid-phase joining technique whereby a bond is formed between two plates by a severe plastic deformation induced by mechanical friction and the heat generated by a rotating tool. Due to the deformation nature of the process, ductile materials such as aluminium [3] are suitable candidates to be processed by the FSW. One of the difficulties with FSW has been the visualisation of microstructure, particularly grain boundaries [4,5]. This is especially a problem for those materials that intrinsically have a fine grain structure. A case in point is aluminium AA6082-T6 [6,7]. This is a marine-grade aluminium alloy [8], with high-strength mechanical properties (Elastic Young’s Modulus of 71 GPa, Fatigue Strength of 95 MPa, Shear Modulus of 26 GPa, Shear Strength of 220 MPa and Ultimate Tensile Strength of 330 MPa) [9], compared to other Al-series. Furthermore, the T6 tempering cycle achieves an artificially aged super-saturated solid solution to meet a high-strength structure compared to other 6xxx-series alloys [10,11]. The standard chemical composition for AA6082-T6 is shown in Table 1. Although this corrosion-resistance Al alloy is a suitable choice for machining, it suffers from poor weldability [8]. It has historically been difficult to demonstrate the microscopic features for this material, which has hindered the diagnosis of the causes of its poor weldability [12].

Recent novel developments have yielded an etchant that is capable of showing microstructures using optical microscopy (OM) [5]. Applications of the etchants have elucidated the grain size and morphology in different regions of the weld texture [7], however, the grain boundary network and thermomechanical features (e.g., dynamic recrystallisation evolution and grain refinement mechanisms) need more advanced and precision measurement such as electron microscopy [13,14]. There is also a need to compare and contrast the different features evident in the optical and electron methods, and to validate the etchant method. Furthermore, there is a need to better understand the linear features, or flow lines, evident in the cross section.

### 1.2. Background Literature

Due to the severe shear deformation during friction stir welding, it is not straightforward to evaluate the microstructure evolution of the FSW weld [16]. The grain map of the crystallographic texture can provide an accurate analysis to investigate the relationship between the microstructure and the thermomechanical characteristics of the FSW process [17,18,19]. However, the rotating nature of the tool during the FSW process makes it different to conventional deformation processes, such as rolling, extrusion, or compression. While the shear-bands in these processes are aligned with the deformation direction, the deformation orientation induced within the texture varies across the weld region, as a function of the position of the rotating tool during the FSW process [19,20].

Most of the published research is focused on the characterisation of the grain structure within the FSW weld structure [21]. Another important research strand has been to better understand the material flow [19,20,22] and this requires visualisation of the texture variations in the aluminium FSW welds [17,18,19].

Furthermore, due to differences in heat generation [23] and flow mechanism [24] between FSW and BFSW, the texture evaluation is expected to be different. This has been observed in the microstructural evolution of the AA6082-T6 BFSW weld structure [4,5]. The flow arms at the hourglass-borders of the BFSW weld [25] are different to the onion ring patterns in the basin-shaped FSW weld structure. Therefore, these two welding processes show different features, and there is a need to better understand the dynamic recrystallised grain structure and flow-based characteristics of the weld region.

### 1.3. Approach

The present paper compares the optical microscopy results (using etchant), against electron backscatter diffraction (EBSD) results to analyse the bobbin friction stir welding (BFSW) weld texture of aluminium alloy AA6082-T6 with a focus on the thermomechanical details of the microstructure.

EBSD analysis was used to determine the DRX details. This also allows the flow features to be identified at greater resolution compared to OM. Additionally, the grain boundary network and the content of the grain orientation within the weld texture can be investigated with EBSD.

It should be noted that although the transmission electron microscope (TEM) is capable of resolving the fine feature of the microstructure of FSW welds, this research focused on EBSD analysis because of the accessibility and ease of sample preparation.

## 2. Materials and Methods

The AA6082-T6 aluminium plate of 4 mm thickness was used as the workpiece material for the welding trial.

**Figure 1:**
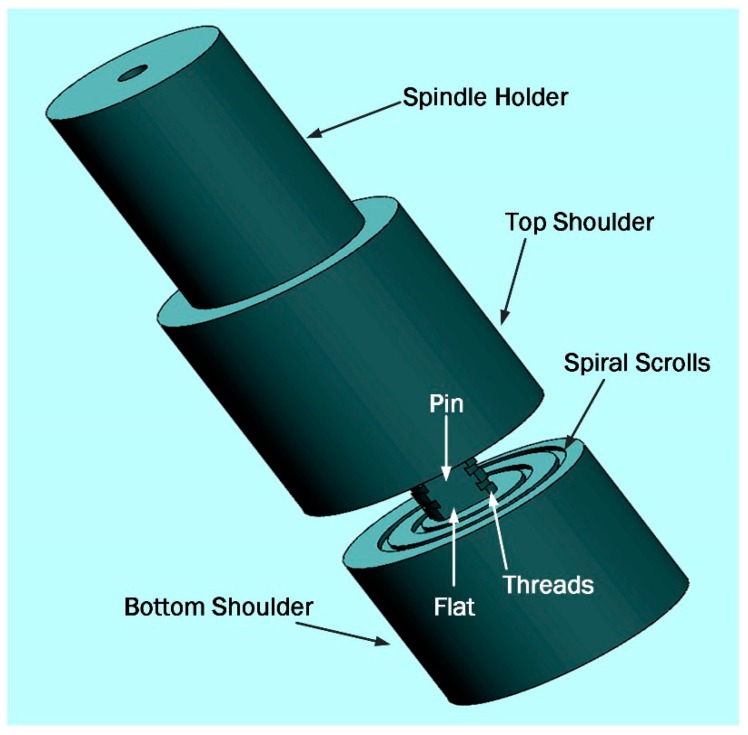
Schematic of the fully-featured Bobbin-Tool (tri-flat threaded pin and spiral scrolled shoulders).

A fixed-gap bobbin-tool, manufactured from H13 tool steel was used for the BFSW welding (Figure 1). The tool was fully-featured (threaded tri-flat pin, and 360-degree spiral scrolled shoulders) to create a better stirring flow. The weld was arranged in the butt-joint position no gap between the plates, also no preheating. A 3-axis CNC machine (2000 Richmond VMC Model, 600 Group brand, Sydney, Australia) was used for the welding trial, while the plates were rigidly fixed by strap clamps at the outer faces during the process.

Since the aim of the research was to evaluate the weld texture in a defect-free structure, a variety of welding speeds (rotational speed; *ω* and advancing speed; *V*) were used to validate the welding process. After running some tests in *ω* (350–650 rpm) and *V* (300–400 mm/min) [5,7,12,26], the optimum welding trial was performed in clockwise rotational speed (*ω*) of 600 rpm with an advancing speed (*V*) of 400 mm/min in the traveling direction. This set of speeds (*ω*, *V*) achieved a weld with no crack or void defect on surface, neither any material loss through the weld-seam. Lower speeds were unable to create a bonded weld between the aluminium plates, and higher speeds deteriorated the quality of the weld by material loss. The details of the welding test are listed in Table 2.

After conducting a 150 mm single-pass weld line, the sample was cut along the transverse direction of the plates for metallographic analysis.

The AA6082-T6 weld samples were prepared first for etching, and then repolished for EBSD. On both cases the polish method was per: standard mechanical polishing with different grades of SiC sand papers (600-grit, 800-grit and 1200-grit). To achieve a mirror surface, the micro-polishing step was conducted on a micro-cloth pad with a 3 μm diamond paste, and finally a 0.05 μm colloidal silica solution [5].

The samples then were etched in a reagent etchant solution with the composition of (0.5 g (NH_4_)_2_MoO_4_ + 3.0 g NH_4_Cl + 1 mL HF + 18 mL HNO_3_ + 80 mL H_2_O). The immersion etching was done in an ultrasonic bath for 90 s, at 70 °C. The microstructure of the etched cross-sections was examined by optical microscopy (OM).

Samples were repolished back to 600-grit between optical and EBSD examination. Based on sizes of polishing particles, this might correspond to about 100 μm surface removal. Our experience in repolishing for optical work shows that flow features are reasonably consistent after repolishing. Samples were positioned by geometric measurements from the edges of the weld, in such a way to view the same region of the weldment. Repositioning accuracy is estimated to be with 20 μm. For both these reasons the repolished surface features evident in the results may not correspond exactly to each other in the pairs of images.

For EBSD the following process applied: the mounted specimens were examined with a scanning electron microscope (SEM) (JEOL 6100, JEOL Inc., Peabody, MA, USA) with an HKL Nordlys III EBSD detector (Oxford Instruments plc, Abingdon, UK). The EBSD plots for different regions of the weld region were reconstructed with HKL Tango software (HKL Channel 5 Tango software version 5.12.60.0, Oxford Instruments plc, Abingdon, UK) [27].

For the mid-Stirring Zone (SZ) region with the ultrafine grain structure, a magnification step size of 0.75 μm with an overall acquisition area of (~400 × 300 μm) was used for EBSD mapping. Alternatively, for other regions of the weld with larger grains, a step size of 3 μm with an overall acquisition area of 2 mm^2^ was used, while all other control parameters such as binning, probe current, accelerating voltage, and exposure time were held constant [7,27]. The average indexing rate of all collected samples was 97.5%, where the unindexed pixels were filled in with the software [28].

Using the average Taylor factor, the EBSD data was further analysed for the distribution of the grain boundaries within the microstructure [29,30]. The low-angle grain boundaries (LAGBs) with misorientation degree of 2°–10° were highlighted in blue and the high-angle grain boundaries (HAGBs) with misorientation degree larger than 10° were shown by red [7,31]. It should be noted that the twinning boundaries were not analysed but instead included in the LAGBs distribution map.

## 3. Results

### 3.1. Characterisation of the Sample Regions with OM

**Figure 2:**
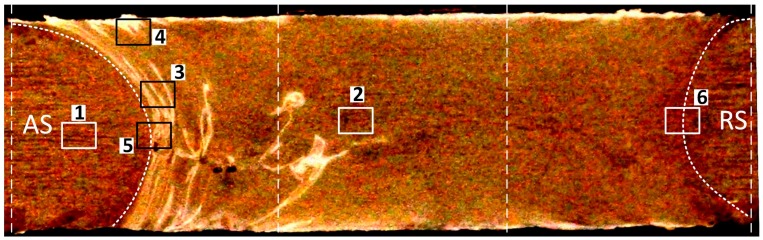
Weld cross-section of the AA6082-T6 plates, (1) Base Metal; (2) Middle Stirring Zone; (3) Flow-arms; (4) Sub-shoulder region; (5) Advancing Side (AS) Hourglass border and (6) Retreating Side (RS) Hourglass border. The two dashed-lines in the middle are representative of the position of the pin, the dashed-line in the corners of the cross-section are representative of the width of the shoulders.

The samples were from various areas of the weld cross-section as described below and in Figure 2. The cross-section is perpendicular to the welding direction as the Advancing Side (AS) of the weld region is situated in the left and the Retreating Side (RS) is on the right at the cross-section.

1. Base metal (BM). This is the parent metal of the workpiece and situated outside of the weld region far away from the active region of the stirring, and thermally and mechanically unaffected by the welding process. In the AA6082-T6 workpiece it is expected that BM would conserve the columnar-shaped directional grain morphology of the rolled structure with the primary average grain size unchanged during the BFSW welding process, and this is what was observed.

2. AS/RS Hourglass Borders. The interfaces between the plastic deformation region or Stirring Zone (SZ) and the transition region adjacent to the weld region are distinguished as hourglass shaped borders at both the AS and RS of the weld. The bent shape of the border at the middle of the cross-section can be attributed to the interaction of the pin and shoulders with the substrate. The pin induces more shear compared to shoulders, therefore the borders are stretched towards the pin position. The grains size and morphology in the texture of hourglass border is observed to be different than both BM and SZ. This is due to the different thermal and mechanical characteristics through the cross-section.

3. Flow-arms. These are characteristic microscopic feature of the BFSW weld structure, evident as elongated bands in direction of the stirring flow lines from the centre of the weld drawn towards the top and bottom shoulders. The formation of the flow-arms is generally attributed to a direct outcome of the shear banding in a continuous plastic deformation, where the layered mass flow is deposited by the advancing of the rotating tool.

4. Sub-shoulder region. A severe plastic deformation is experienced underneath the shoulders. The scrolled features of the shoulders may increase the frictional heat generated in this region of the weld. Study of the sub-shoulder texture in microscopic scale can potentially reveal thermomechanical details of the BFSW process as the thermal and mechanical stress/strain fields are in a maximum rate in this region.

5. Mid-SZ. This is the main region of the weld represented by the ultrafine equiaxed grain texture in comparison with the BM. As will be shown below, the Mid-SZ region experiences full dynamic recrystallisation (DRX) transformation including grain refinement and precipitation, more than any other region in the weldment. However, because of the specific ultrafine characteristics of the microstructure of the SZ, a precise micro-analysis of the Mid-SZ texture requires electron microscopy rather than the OM metallographic measurements.

### 3.2. Texture Evaluation with EBSD

The crystal orientation texture results from the EBSD mapping should be referenced compared to the RD-TD-ND perpendicular directions as a reference frame. In BFSW welded plates, the proprietary EBSD parameters need to be redefined in TD-ND-WD direction frame, as:

- Transverse direction (TD); perpendicular to the welding direction, parallel to the cross section of the weld, where the AS is in (−) and the RS is situated in the (+) of the TD axis.
- Normal direction (ND); perpendicular to the plate surface, representative of the distance between the top and bottom surface.
- Welding direction (WD); the direction of the advancement of the tool, parallel to the weld-line.

**Figure 3:**
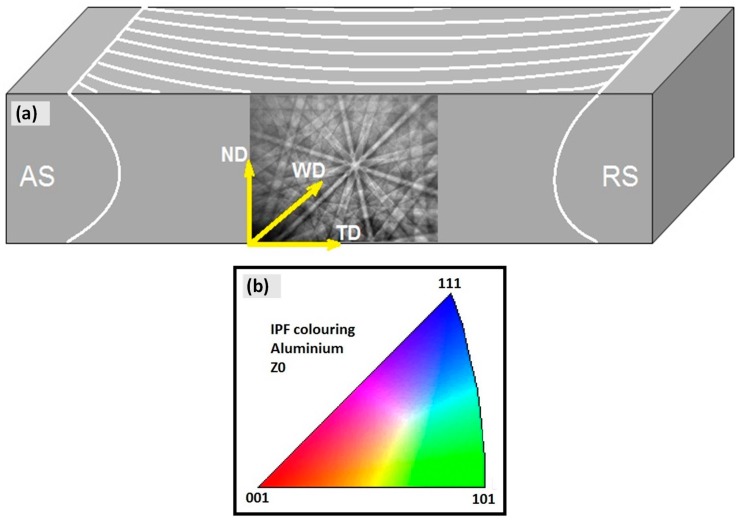
Crystallography directions in Aluminium alloys, (**a**) Schematic of ED, ND and TD reference directions, applied for EBSD analysis; (**b**) IPF colour triangle. (Source for (**b**) Ex EBSD machine).

For the texture observation of the AA6082-T6 BFSW weld, the grain maps were indicated in the TD-ND plane for different regions of the weld cross-section. This plane is identical to the cross-section etched for the optical microscopy. The directions of the orientation used for the texture analysis of the EBSD plots are shown in Figure 3a.

The maps from the EBSD analysis present the following microscopic information: grain orientation map and grains boundary map. In the first phase, the colour mapping based on the crystallography directions of the grains indicates the crystal orientation map distribution for the analysed region. For the EBSD the standard inverse pole figures (IPF) map was used per Figure 3b. This shows the three main crystallographic directions within the grains distinguished by different colours; red for (001), green for (101) and blue for (111).

The colours in Figure 3b show the corresponding orientation of grains and crystals with respect to (001), (101) and (111) orientations. While the main crystallographic orientations are demonstrated by these three colours of red, green and blue, the other crystallographic directions between these three main crystallographic orientations are shown with the mixed colours. To obtain the grain boundary misorientations it would be necessary to specifically measure the individual misorientation.

In the second phase of the EBSD analysis, the grain boundary maps were derived in the post processing data procedure to measure the density of the high-angle grain boundaries (HAGBs) and low-angle grain boundaries (LAGBs) within the texture. Similar to the grain misorientation mapping, the HAGBs were defined by a misorientation angle greater than 10° and LAGBs were defined as the misorientation angles between 2° and 10°.

In some analyses 15° is used as the LAGBs/HAGBs transition demarcation rather than 10°. This is because at such higher angles the misorientation is more definitive to show the grain boundaries. Nonetheless the misorientation angle between 10° and 15° also can be counted as the HAGBs.

The following sections compare the results from the optical and EBSD approaches.

### 3.3. Base Metal

**Figure 4:**
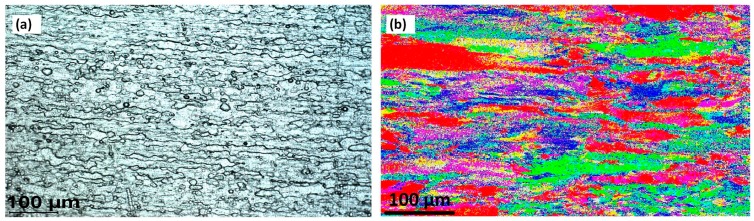
Base Metal microstructure, (**a**) Optical microscopy and (**b**) EBSD colour map.

The optical and EBSD microscopy results (IPF map) are shown in Figure 4. This shows that the overall structure is represented similarly between the two methods: columnar grains of rolled base material are evident. The crystal direction (grain orientation) is similar. The colours in the EBSD results show that a variety of orientation occurs, and the rolling effect is observable by a columnar directional alignment in the orientation distribution, similar to the OM micrograph. The grain boundaries are evident in the optical results but not in the EBSD. The magnification scale was kept similar to the OM micrograph, and post-processing/zooming of the EBSD map shows the GBs.

To apply a post-processing clean-up of the EBSD maps, the Euler map is presented below, with a common orientation in each grain to better view the grain details. However, we followed the same EBSD IPF mapping for the weld region, to compare the similarities of the weld texture with OM micrographs.

### 3.4. Stirring Zone

**Figure 5:**
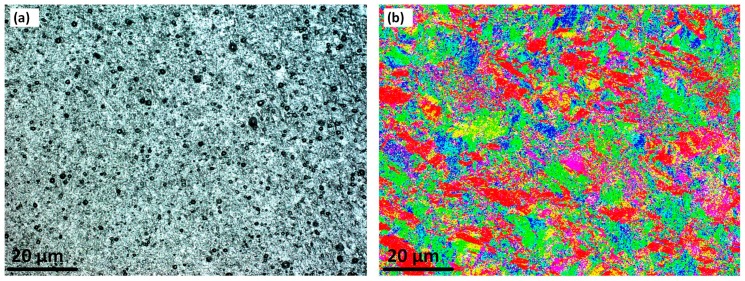
The microstructure of the Mid-Stirring Zone (SZ) region, (**a**) Optical microscopy and (**b**) EBSD colour map.

The stirring zone microstructure for optical microscopy and EBSD is shown in Figure 5. EBSD verifies (which was not possible with OM) that the grain size average is below 10 microns. The randomly distributed grain with equiaxed morphology is evident by the EBSD map. The EBSD IPF colour map (Figure 5b) also demonstrates the microscopic distribution of the misorientation through the SZ texture, in which is not observable via the OM micrograph. The grain boundaries pattern is still bulged which may be related to the ultrafine grain size within the SZ.

### 3.5. Flow Layers

**Figure 6:**
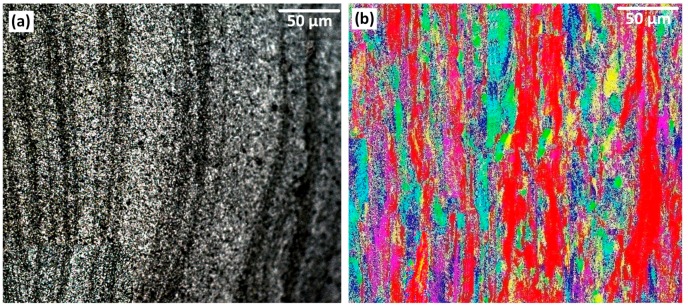
The microstructure of Flow layer patterns, (**a**) Optical microscopy and (**b**) EBSD colour map.

The elongated micropattern of the flow-arms region was compared by optical microscopy and EBSD method (see Figure 6). The continuous flow direction in Figure 6b in red colour is situated at the position of each flow-arm, confirming that each flow-arm possess a specific crystallographic direction compared to the neighbouring region. These macro-bands and macro-regions were readily detected by the EBSD detector and is also present in the etched sample. There is some distance between each branch of flow-arms which has been filled by other crystals with different orientations (distinguished by corresponding colours). In this regard, our interpretation is that the macro-regions of shear bands are aligned along the (001) orientation, in a background of grains with a variation in crystallographic orientation.

This is attributed to the discrete transport mechanism whereby packets of stirred material are deposited at the trailing edge of the tool. In turn this is attributed to the interaction between thread-flat features of the pin tool with the substrate material.

### 3.6. Heat Flow (Sub-Shoulder Region)

**Figure 7:**
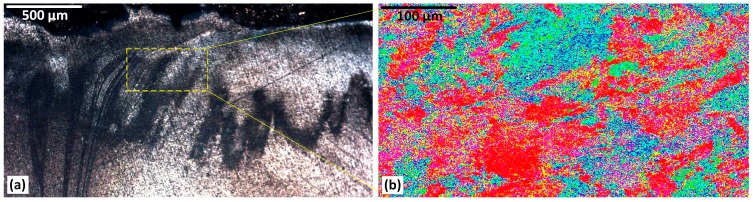
The microstructure of the Sub-shoulder region, (**a**) Optical microscopy and (**b**) EBSD colour map.

The microstructural analysis of the sub-shoulder region is shown in Figure 7, for OM and the related EBSD mapping. The optical microscopy result of the sub-shoulder weld region (Figure 7a) demonstrates the shoulder induced flow. These flow layers represent a transverse transportation of the mass, caused by the frictional behaviour of the shoulder action. Although the microstructure shows a severe plastic deformation underneath the shoulder, it does not contain any specific surface defect. This is attributed to the high compaction effect induced by the direct contact between the shoulder and the top surface of the weld region. The flow patterns are not observed in the deeper areas. This may be representative of the decrease of the compaction effect of the shoulder, and the decreasing effect of the surface shear strain. The flow of the mass at the deeper regions of the stirring zone is more affected by the pin action.

The selected area from the sub-shoulder region shown in Figure 7a has been mapped by the EBSD analysis in Figure 7b. The crystallographic texture at the sub-shoulder region is complex. This is attributed to the complex combination of thermal and strain effects. The approximate position of the flow patterns, delineated in Figure 7a, can be seen in a colour map in Figure 7b. The random dispersion of the grain orientation can be related to the complexity of the strain history in the location, as flow patterns are not evident. However, it should be noted that based on the EBSD standard IPF contouring (colour triangle, Figure 3b), the (001) crystallographic direction (red colour) is more pronounced compared to other orientations. This is the preferred crystallographic direction for the shear planes in the lattice of the Al atoms. Therefore, we interpret this as the shoulder-induced stirring action activating a network of the shear planes in the (001) direction.

### 3.7. Hourglass-Border (AS)

**Figure 8:**
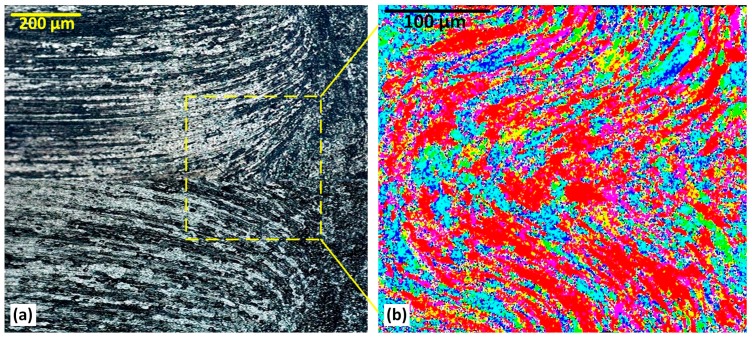
The microstructure of the AS Hourglass border, (**a**) Optical microscopy and (**b**) EBSD colour map.

The highly deformed region at the hourglass-border of the weld as the triple junction is characterised in Figure 7 and Figure 8 for the AS and RS of the weld region, respectively. As shown in Figure 8, the tapered-shape triple junction at the middle of the AS hourglass-border is characterised by a highly deformed elongated grain structure of the base material, stretched towards two opposite directions parting in the middle upwards and downwards. This deformed structure is identified with an upward-downward flow pattern (Figure 8a).

The EBSD analysis of the tip of the tapered-shaped triple junction reveals more details regarding the deformed crystalline structure at the outer part of the hourglass border. The grain orientation of the texture shows the grain structure to be elongated towards the top and bottom shoulders. This micro-pattern is interpreted as DRX features of the FSW process whereby the stored strain induced by plastic deformation affects the grain structure of the TMAZ by grain misorientation. This can be detected as the microscopic change in etching response in the optical microscopy (Figure 8a), and the grain misorientation alteration in the EBSD colour mapping.

The EBSD pattern (Figure 8b) identifies a preferred crystallographic orientation for the deformation-induced grains structure by the red colour representative of the (001) direction from the EBSD standard triangle (Figure 3). Similar to the sub-shoulder region (Figure 7), we identify the red colour as the preferred crystallographic orientation of the Al-based texture for the formation of the shear bands undergone the plastic deformation.

### 3.8. Hourglass-Border (RS)

Similar to the AS hourglass-border in Figure 8, the RS of the weld also has a particular transition appearance at the hourglass-border, where an abrupt alteration in microstructure is visible at the TMAZ.

The delineated micropattern in Figure 8a reveals an ellipse-shape feature as a separating boundary between the TMAZ and the hourglass-border. This structure shows elongated grains at the edge of the TMAZ. This is not stirred material. The effect presumably results from the strain induced by the rotational movement of the tool and the frictional heating.

**Figure 9:**
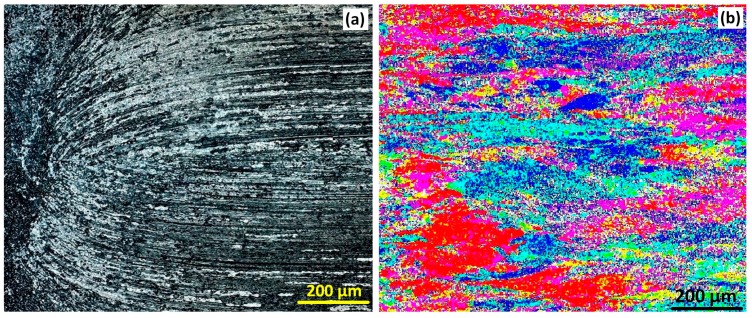
The microstructure of the RS Hourglass border, (**a**) Optical microscopy and (**b**) EBSD colour map.

The micrograph showing in Figure 9b exhibits the EBSD colour map for the ellipse-shape region at the RS, corresponding to the OM microstructure in Figure 9a. It is noted that the EBSD colour map, which shows grain orientation, does not show the same grain morphology apparent in the OM microstructure. Evidently orientation and morphology are decoupled in this region, presumably because of partial recrystallisation. Partly as a result of this somewhat unexpected outcome, we explored the grain angle boundaries in further detail (see the next section).

### 3.9. LAGBs and HAGBs (in the Weld Region)

**Figure 10:**
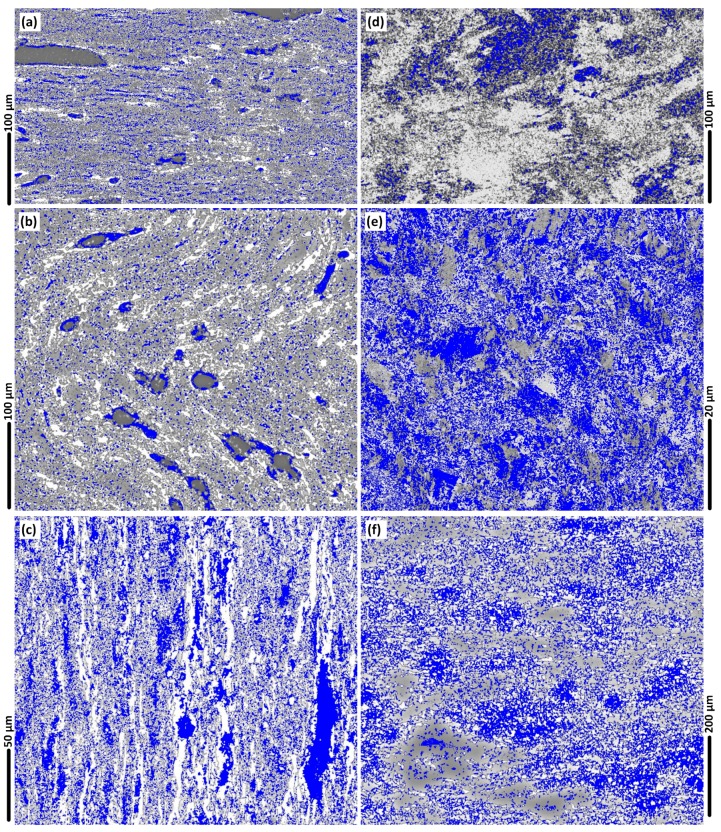
The micropatterns of the presence of low-angle grain boundaries (LAGBs, for 2°–10°) (**a**–**f**) in blue colour, for different regions of the weld; Base Metal (BM) (**a**); AS Hourglass border (**b**); Flow-arms (**c**); Sub-shoulder region (**d**); Mid-SZ (**e**); RS Hourglass border (**f**).

**Figure 11:**
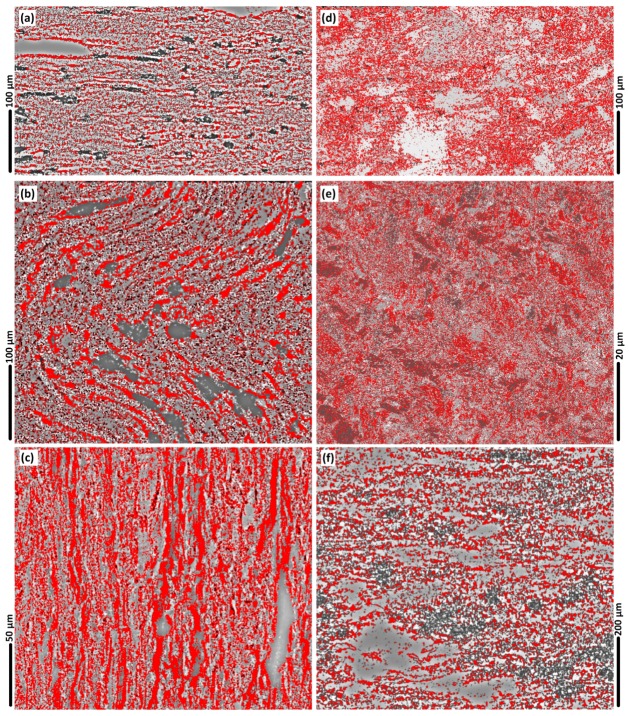
The micropatterns of the presence of high-angle grain boundaries (HAGBs, more than 10°) (**a**–**f**) in red colour, for different regions of the weld; BM (**a**); AS Hourglass border (**b**); Flow-arms (**c**); Sub-shoulder region (**d**); Mid-SZ (**e**); RS Hourglass border (**f**).

The grain boundary mapping for different regions of the weld are shown in Figure 10. The microscopic observations in Figure 10 were processed based on the colour identification for the LAGBs and HAGBs, identified as blue colours (Figure 10) and red colour (Figure 11), respectively.

In the stir zone of Figure 10e the density of the blue colour maps is high, indicating the dominance of LAGBs. Our interpretation is that the LAGBs are representative of the position of the sub-grain boundaries or precipitates, which are a direct result of the DRX.

The comparison between the grain orientation maps and grain boundary maps indicates that the position of the LAGBs is aligned with the position of the crystallographic misorientation inside the grains. This is more evident in the AS of the weld or the mid-SZ region where the texture experiences severe plastic deformation compared to the rest of the weld region.

For a better understanding of the BFSW weld texture, the EBSD Euler contrast maps were constructed by post-processing of the EBSD data to denote the overlaid grain boundaries with a transition misorientation angle of 15°.

**Figure 12:**
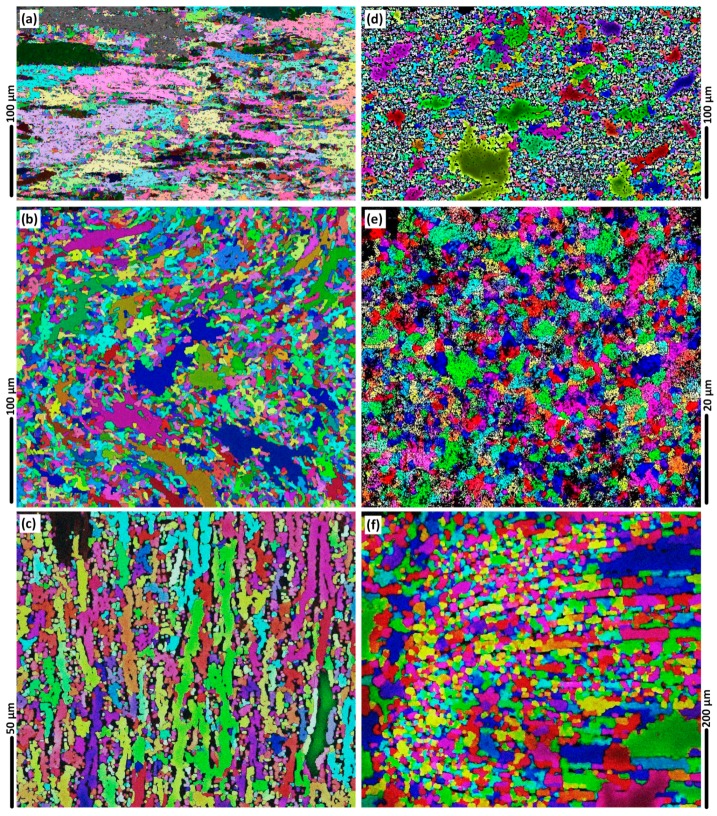
The Euler map micropatterns of the presence of HAGBs with post processing using a 15° angle, for different regions of the weld; BM (**a**); AS Hourglass border (**b**); Flow-arms (**c**); Sub-shoulder region (**d**); Mid-SZ (**e**); RS Hourglass border (**f**).

Assuming a 15° threshold for grain boundary elucidation, gives the results shown in Figure 12. Results emphasise the display of the HAGBs in the Euler orientation map. Precipitates are more prominent in these views compared to the previous IPF maps. Otherwise the morphology and shear bands show the same outcomes as before, indicating that the results are insensitive to choice of threshold angle. Moreover, compared to IPF maps, the Euler maps are more limited in ability to detect small orientation changes of the LAGBs, hence these are not distinctly observable.

## 4. Discussion

### 4.1. Comparison between Methods

When applied to the BFSW welding case, the main benefit of optical microscopy (with etchant) is that it shows the size of the grains, i.e., the morphology. It does so at relatively low cost. It should be noted that the optical microscopy micrographs delineate the grains by the same colour without any information regarding the distribution of the crystallographic orientation or lattice structure. Moreover, the AA6082-T6 is difficult to obtain sufficient contrast in OM, and depends on suitable reagents and etching procedures.

A better understanding of weld texture arises from application of EBSD, which shows the grain orientation. This is useful because it shows that adjacent grains often have very different orientations. This is attributed to the stirring action. The EBSD map can show the grain orientation distribution of the sample, illustrating the morphology and size of grains with a precise accuracy constructed with the spatial digital pixel derivation. Based on the EBSD mapping analysis, the neighbouring pixels with similar crystallographic orientations are representative of a grain region, identifying by the same colour.

The study verifies that the method of optical microscopy with etchant gives results that are consistent with EBSD, though naturally the type of information available is different. There are advantages and disadvantages to the two methods (see Table 3).

Another powerful instrument for characterisation of the DRX detail and grain growth structures within the weld region is the transmission electron microscope (TEM) [32]. Specifically, TEM also enables detection of precipitation phenomena, often intermixed with the dislocation network and the grain boundaries structure [33,34]. This might elucidate the solid-state plastic flow mechanism in the BFSW processed structure of the AA6082-T6 in correlation with the microstructural effect of the T6 temper. This is left for future research, as a TEM study is beyond the scope of this paper.

### 4.2. Microstructure of Welded AA6086-T6

In this work, we present a metallographic measurement (by OM and EBSD methods) to elucidate some microstructural performance of the AA6086-T6 weld texture, dominant by the thermomechanical nature of the BFSW process.

#### 4.2.1. Shear Bands

From the EBSD colour orientation mapping, it can be concluded that the (100) red and {111} <101> turquoise are the dominant orientations at the hourglass borders, indicated by a layered/fibrous texture.

After the severe shearing, the polycrystalline lattice transforms to the zero-distortion planes by the displacement. The most plausible shear plane with the minimum stacking fault energy in FCC lattice is (100)//WD. Moreover, during the DRX, the slip system of {110} <111>//TD activates the rearrangement of orientation which is revealed as turquoise colour. This is a diffusive transformation to stabilise the interface energy between the shear texture of (100) plane with the shear strained region at its immediate proximity to obtain a coherent interface.

The grain orientation maps of the BFSW sample confirmed that the shear texture at the AS interface of the weld region indicates a more abrupt transition compared to the RS. The occurrence of the discontinuity defects (e.g., tunnel void) occurs at the AS, rather than the RS. Based on this observation, our interpretation is that the texture characteristics in BSW weld correlate with a location-dependency microscopic evolution, where the shear-induced locations (due to the rotation and advancement of the tool) can increase the potential of failure within the weld structure.

The relative motion between the directions of the rotation and advancement of the tool are the same at the AS, which results in an elevated rate of flow (directed forward) and hence plastic deformation. On the other hand, because of the opposite directions of the rotation and advancement of the tool at the RS, the stirred mass is compacted at the trailing edge of the tool, leading to the mixing of the layers (leading to lower level of distinction between the layers in metallographic observations), and refilling of the possible discontinuities (no void formation observed in the RS).

The elongated-shape texture at the hourglass border was studied in TD-ND plane of the EBSD plots. However, because of the dependency of this texture to the (100) shear plane, it needs to be further studied in ND-WD plane through the crystal direction aligned to both ND and RD. These directions may better identify the shear at the circumferential of the tool for the deposited layers along the AS.

The crystallographic nature of these shear band textures may be worth further study. The thermomechanical behaviour of the BFSW and semi-solid plastic deformation are possible explanations for the orientation evolution of the shear texture at the hourglass border interface.

The (100) shear direction in the FCC lattice can be activated when the uniaxial hot deformation compression happens to the texture. The microscopic observations suggest that the high mobility of the grain-boundaries during the deformation results in formation of elongated grains within the (100) shear texture.

In the growth competition between different crystallographic directions, the (100) planes provide a preferential axis for the faster grain-boundary migration. Hence the red (001) material is frequently surrounded by turquoise material {111} <110>. Turquoise is also sheared material, but in the orthogonal orientation. This indicates that there is an element of secondary alignment of grains alongside the main (red) shear bands. Some possible interpretations are that the turquoise material represents material that is compressed between the shear bands. There are also pink and yellow regions, but blue and green are sparse.

During the recovery and DRX after the process, it is assumed that the stored strain and the temperature can provide the driving force to activate other crystallographic systems to change the texture. However, because the elongated grains from the (100) system were formed already during the mechanical stirring process, the similar structures in their proximity has more preference. In the case of FCC metals, the crystallographic system of {111} <110> is a stable orientation for the recovery of the plane-strain compression deformed texture during the recrystallisation. This is the turquoise colour regions in EBSD plots, which is situated between the (111) and (101). In OM these shear bands are evident as dark lines. These lines comprise layers that are sheared and stacked in directions aligned with the shear flow changes.

Shear causes the grains to be compacted (as evidenced in the red (001) orientation). By definition this means fewer stacking faults, and fewer dislocations. The EBSD shows that the shear effect is layered—there is no uniform shear across the section, though there is similarity of orientation between adjacent sheared areas. This has potentially significant implications for the modelling of internal flows via computational fluid dynamics (CFD) modelling, where it is assumed that the flow is locally consistent. In the BFSW samples under examination, the shear occurs in discrete layers. This may have something to do with the transport of material around the pin, in a type of packet flow.

#### 4.2.2. Internal Flow

The homogeneity of the grain structure and the random distributed grain refinement took placed in the SZ (as the middle of the weld region) represent a simultaneous grain fragmentation and severe plastic deformation during the FSW process and the subsequent DRX taking place by the re-cooling, respectively.

The microscopic comparison between the sharp tapered-shape boundary at the AS and the ellipse-shape boundary at the RS may represent a difference in rate of the torsion for the two zones. This can be also related to the different directions of the tool motion and the mass transportation, by which causing different strain rates and the subsequent shearing patterns. Furthermore, different temperature gradient in the AS and RS can directly influence the DRX mechanism subjected to the frictional heating.

The elongated grains as the flow-arm region was characterised as a periodic deformed structure caused by both deformation and thermal load induced by the pin-driven stirring action. Additionally, underneath the shoulder, a continuous plastic flow appears which forms the sub-shoulder region. The microstructure in the sub-shoulder region is slightly coarsened compared to the internal parts of the SZ. This may be related to the cooling conditions experienced by the surface of the workpiece in free contact with the air as the coolant.

The triple flow junction at the middle of the hourglass-border is attributed to the two different characteristics of the flow there. The middle part of the weld region experiences severe thermo-plastic flow regime driven by the stirring action of the pin, which appears to provide two flow path ways towards the upper and lower parts of the cross-section.

### 4.3. Implications: Towards an Interpretation of the Interaction between Physical Metallurgy and Flow

Based on the theory of FSW, the alteration of the texture in different region of the weld (SZ, HAZ, TMAZ) was expected. However, the present work elaborates on the structures and DRX mechanisms for the BFSW case. Shear bands features were identified in different microscopic details through the hourglass-borders and the sub-shoulder region. These can be explained by the induced strain and thermal history.

In general, the microscopic observations confirmed that the deformation induced by the rotating BFSW tool is typically in the form of shear. However, due to the physical rotation of the tool, the shear texture shows different local orientation throughout the weld texture. Moreover, it should be noted that the shear flow layers within the weld are affected by the depth of the material as the position of the pin and shoulders can induce different local shearing modes. The shear layers are not necessarily aligned with the weld direction. This can be due to the rotating nature of the tool. For example, even in the middle of the SZ, the equiaxed grain texture shows the minimum of anisotropy within the sample, representative of the uniform distribution of the shear. For the processing of the aluminium alloys, the EBSD texture observations should be compared to the possible shear planes reported in the literature for the face-cantered cubic (FCC) crystallographic unit: red for (001).

The absence of the precipitate phase in the microstructure observations for both of OM and EBSD results implies that the BFSW welding happened without precipitation. However, this is not usual for a dynamic recrystallised texture that experienced a severe plastic deformation accompanied with the frictional heat generation. Possibly this may be related to the scanning mode of the microscope, and hence this may need to be checked in future studies.

The formation of the shear bands is related to the details of the mechanical stirring action. Our explanation is as follows. By the contact between the rotating tool and the workpiece, the primary grains located at the advancing edge of the tool undergo fragmentation and subsequent plasticising by the frictional heat and deformation. The simultaneous rotation and advancement of the tool squeeze the softened grains to the trailing edge of the tool. This causes a bending and elongation yielding through the morphology of the grains which are deposited at the AS and RS sides of the tool, where the shear is maximum. However, at the RS because of the opposite directions of the rotation and translation, the flow layers are mixing together, and the elongated grain structure is less clear. Therefore, the hourglass interface between the TMAZ and the SZ on the AS is more observable rather than the RS. In other words, the plastic flow on the AS swept in a smooth narrow shear band at the proximity of the pin, while the irregular transformation of the flow at the hourglass-interface on the RS cause a kind of mixing of the shear-bands. At the AS-RS region behind the tool, the stirring action breaks up the transformed grains into the ultrafine equiaxed grains.

The coarsened and elongated grain textures at the AS and RS compared to the mid-SZ equiaxed grains can be attributed to the multi-directional strain-induced flow fields, caused by the top and bottom shoulders. While in mid-SZ region, pin-induced shear appears to dominate the plastic flow, and the subsequent stored-strain becomes the driving force of the DRX. Therefore, the fully DRX in mid-SZ produce an ultrafine grain structure compared to the AS and RS interface regions, because of a higher rate of stored-strain driven by the direct action of the stirring pin. Moreover, at the sub-shoulder region, the ultrafine DRX grains are intensified by the severe frictional effect of the shoulder and the subsequent thermal dissipation due to the cooling by the free air surrounding the weld surface. Therefore, the sub-shoulder region shows a different flow pattern compared to the mid-SZ region and the hourglass-borders.

Based on the ultrafine equiaxed grain structure, it is proposed that the mid-SZ grains have undergone a severe DRX. In contrast the elongated grains in the transition region at the hourglass interface of the weld region indicate a different morphology implying a local deformation and recovery with partial DRX.

The literature explains that the stirring action is directly responsible of the formation of the shear texture. In the case of BFSW the shear deformation around the pin is non-uniform with a thermal and strain gradient across the weld. Hence in BFSW welds there is an inherent flow mechanism (induced from the pin and shoulder features), which transports the stirred mass around the weld in discrete packets. This induced shear, which was followed by a DRX process (partial or full depending on the shear), which led to the observed polycrystalline structures (and grain boundaries).

Regarding the texture evolution it should be noted that beside the shearing deformation, the frictional heat input (generated by the interaction between the tool-workpiece) has a key role in microstructural alteration during DRX. Similar to the induced strain, the variation in thermal flux from the position of the pin (mid-SZ) to the top and bottom shoulders, causes local heating on the deformed material. Beside the stored strain, this heat input contributes to accomplish a fully-DRX through the mid-SZ. Other regions of the FSW weld, such as hourglass-borders, TMAZ and HAZ, also are affected by heat, but to a lesser extent. Hence the thermal contribution to the DRX varies according to the location in the weld. Temperature also has a direct effect in formation of LAGBs/HAGBs and precipitation during DRX in different regions of the weld.

Welding process settings (workpiece thickness, tool geometry, welding speeds) are expected to affect the microscopic details of the weld texture. Increase in the thickness of the workpiece may reduce the generated heat and shrink the thermal flux, hence decreasing the DRX process. However, this might possibly be compensated by more complexity in tool geometry to induce more flow through the stirring action. Similarly, increase in welding speeds (*ω*, *V*) is expected to increase the frictional heat at the position of the tool-material, resulting in a more severe plastic deformation. Therefore, the DRX mechanism can potentially be intensified by suitable optimisation of the welding parameters. This might be worth investigating via microscopic observation to identify the optimum settings.

### 4.4. Future Work

The 6-series aluminium alloys (Al-Mg-Si) such as AA6082-T6 as here, are generally problematic in FSW processing. It is already known that precipitation occurs during the process [7]. The present study identified that the texture includes shear bands with fibrous morphology in the grain orientation. One of the possible reasons can be work hardening. This is a plausible mechanism based on our observation of the precipitation in Euler maps. However, the characterisation ideally needs to be developed by TEM and in higher magnification to identify the origins of the phenomenon and elucidate the origins of the precipitation mechanism. Hence, we suggest that future work could consider investigating work hardening and anisotropic behaviour of the texture during stirring and DRX.

## 5. Conclusions

The study verifies that the method of optical microscopy of AA6082-T6 BFSW weld with etchant per [5] gives results that are consistent with EBSD, though naturally the type of information available is different. The EBSD results, including grain orientation mapping and grain boundary mapping, reveal more details of the weld texture. The EBSD colour mapping demonstrated the grain misorientation in different regions of the weld, caused by the shear during stirring action and the subsequent dynamic recrystallisation (DRX). The grain boundary mapping, based on the Taylor factor, distinguished low-angle grain boundaries (LAGBs) and high-angle grain boundaries (HAGBs) through the weld texture.

It was evident that different grain orientations were formed by the shear strain induced by the tool performance in AS and RS of the weld. The hourglass-border as a direct outcome of the shearing was formed as the aligned bands at the AS, while the complex action of the tool caused the mixing of shear bands at the RS.

The grain structures that occur in AA6082-T6 BFSW welds are due to a complex set of factors. The motion of the pin and shoulder features transports material around the weld, which induces shear. The shear deformation around the pin is non-uniform with a thermal and strain gradient across the weld, and hence the DRX processes are also variable, giving a range of polycrystalline and grain boundary structures.

This micro-pattern is interpreted as DRX features of the FSW process whereby the stored strain induced by plastic deformation affects the grain structure of the TMAZ by grain misorientation. This can be detected as the microscopic change in etching response in the optical microscopy (Figure 8a), and the grain misorientation alteration in the EBSD colour mapping.

This paper makes the original contribution of identifying the content of the flow lines that are evident in the macro-structure. These lines are evident in optical microscopy, but the images in the literature are typically diffuse due to the difficult of etching this material. As shown here, with suitable etchants OM shows the grain boundary features more clearly. More accuracy is evident with EBSD, which also avoids risks of introducing artefacts due to over-etching. The results show the OM and EBSD results are consistent. The overall interpretation is that the welded region contains fine discrete layers of material, of approximately 10 μm thickness. In these the shear has caused dynamic recrystallisation, which has resulted in a string of grains with similar orientation. When etched and observed with OM, these appear as dark and light lines.

Furthermore, these shear bands are found to have different characteristics across the weld section. In turn this is attributed to the different flow and thermal regimes in these locations. A homogeneous continuous fluid would be expected to have a smooth shear field. The actual observer shear occurs in discrete bands. This, plus the abrupt changes in the lines, suggests that the internal flow regime is more complex than might be expected.

## Figures and Tables

**Table 1:** Element composition of the AA6082-T6 aluminium alloy (wt %) [15].

Chemical Element	Present (wt %)
Silicon (Si)	(0.70–1.30)
Magnesium (Mg)	(0.60–1.20)
Manganese (Mn)	(0.40–1.00)
Iron (Fe)	(0.0–0.50)
Chromium (Cr)	(0.0–0.25)
Zinc (Zn)	(0.0–0.20)
Titanium (Ti)	(0.0–0.10)
Copper (Cu)	(0.0–0.10)
Other (Each)	(0.0–0.05)
Other (Total)	(0.0–0.15)
Aluminium (Al)	Balance

**Table 2:** The specification of the operation parameters for the AA6082-T6 BFSW weld sample.

Workpiece	Tool Material	Work Temp °C	*D*_Shoulder_ (mm)	*D*_Pin_ (mm)	Plate Thickness (mm)	Feed ω (rpm)	Speed *V* (mm/min)	Thread Pitch (mm)	Number of Threads in the Gap
AA6082-T6	H13 Tool Steel	18	21	7	6	600	400	1.5	4

**Table 3:** Comparative details of the metallographic measurements performed for the bobbin friction stir welding (BFSW) A6082-T6 weld structure by optical microscopy (OM) and EBSD.

Metallographic Measurement	Pros	Cons
Optical microscopy (with etchant)	Grain boundaries visible (but orientation not)	Precipitation not evident
EBSD	Crystal orientation visible. Misorientation between grains is evident	Precipitation not evident at this level of magnification
Combination of both methods	Characterised microscopic features of the BFSW weld by OM, was validated by EBSD. Further details of the shear texture in different regions of the weld were evaluated by EBSD	Due to repolishing, the measurements are time-consuming and it is not possible to repeat the exact position of the microscopic features

## References

[1] Thomas W., Nicholas E., Needham J., Murch M., Temple-Smith P., Dawes C. (1991). Friction Stir Butt Welding. GB Patent.

[2] Thomas W., Nicholas E. (1997). Friction stir welding for the transportation industries. Mater. Des..

[3] Threadgill P., Leonard A., Shercliff H., Withers P. (2009). Friction stir welding of aluminium alloys. Int. Mater. Rev..

[4] Sued M., Tamadon A., Pons D. Material flow visualization in bobbin friction stir welding by analogue model. Proceedings of the Mechanical Engineering Research Day 2017.

[5] Tamadon A., Pons D., Sued K., Clucas D. (2017). Development of metallographic etchants for the microstructure evolution of a6082-t6 bfsw welds. Metals.

[6] Tamadon A., Pons D., Sued K., Clucas D. (2018). Formation mechanisms for entry and exit defects in bobbin friction stir welding. Metals.

[7] Tamadon A., Pons D., Sued K., Clucas D. (2018). Thermomechanical grain refinement in aa6082-t6 thin plates under bobbin friction stir welding. Metals.

[8] Sued M.K. (2015). Fixed Bobbin Friction Stir Welding of Marine Grade Aluminium. Ph.D. Thesis.

[9] Avallone E.A., Baumeister III T. (1986). Marks’ Standard Handbook for Mechanical Engineers.

[10] Aginagalde A., Gomez X., Galdos L., García C. (2009). Heat treatment selection and forming strategies for 6082 aluminum alloy. J. Eng. Mater. Technol..

[11] Mohamed A., Samuel F. (2012). A review on the heat treatment of al-si-cu/mg casting alloys. Heat Treatment–Conventional and Novel Applications.

[12] Sued M., Pons D., Lavroff J., Wong E.-H. (2014). Design features for bobbin friction stir welding tools: Development of a conceptual model linking the underlying physics to the production process. Mater. Des..

[13] Davies P., Wynne B., Rainforth W., Thomas M., Threadgill P. (2011). Development of microstructure and crystallographic texture during stationary shoulder friction stir welding of ti-6al-4v. Metall. Mater. Trans. A.

[14] Tayon W.A., Domack M.S., Hoffman E.K., Hales S.J. (2013). Texture evolution within the thermomechanically affected zone of an al-li alloy 2195 friction stir weld. Metall. Mater. Trans. A.

[15] Davis J.R. (1993). Aluminum and Aluminum Alloys.

[16] Fonda R., Bingert J., Colligan K. (2004). Development of grain structure during friction stir welding. Scr. Mater..

[17] Fonda R., Bingert J. (2007). Texture variations in an aluminum friction stir weld. Scr. Mater..

[18] Fonda R., Knipling K. (2011). Texture development in friction stir welds. Sci. Technol. Weld. Join..

[19] Fonda R., Knipling K., Bingert J. (2008). Microstructural evolution ahead of the tool in aluminum friction stir welds. Scr. Mater..

[20] Fonda R., Reynolds A., Feng C., Knipling K., Rowenhorst D. (2013). Material flow in friction stir welds. Metall. Mater. Trans. A.

[21] Prangnell P., Heason C. (2005). Grain structure formation during friction stir welding observed by the ‘stop action technique’. Acta Mater..

[22] Coelho R.S., Kostka A., Dos Santos J., Pyzalla A.R. (2008). Ebsd technique visualization of material flow in aluminum to steel friction-stir dissimilar welding. Adv. Eng. Mater..

[23] Hilgert J., Schmidt H., Dos Santos J., Huber N. (2011). Thermal models for bobbin tool friction stir welding. J. Mater. Process. Technol..

[24] Hilgert J., Hütsch L.L., dos Santos J., Huber N. Material Flow Around a Bobbin Tool for Friction Stir Welding. Proceedings of the COMSOL Conference.

[25] Hilgert J., Dos Santos J., Huber N. (2012). Shear layer modelling for bobbin tool friction stir welding. Sci. Technol. Weld. Join..

[26] Tamadon A., Pons D., Sued M., Clucas D., Wong E. Preparation of plasticine material for analogue modelling. Proceedings of the International Conference on Innovative Design and Manufacturing (ICIDM2016).

[27] Beardsley A., Bishop C., Kral M. (2016). Ebsd characterization of pilgered alloy 800 h after heat treatment. Mater. Perform. Charact..

[28] Jackson M.A., Groeber M.A., Uchic M.D., Rowenhorst D.J., De Graef M. (2014). H5ebsd: An archival data format for electron back-scatter diffraction data sets. Integr. Mater. Manuf. Innov..

[29] Paul H., Driver J., Tarasek A., Wajda W., Miszczyk M. (2015). Mechanism of macroscopic shear band formation in plane strain compressed fine-grained aluminium. Mater. Sci. Eng. A.

[30] Li R., Xie Q., Wang Y.-D., Liu W., Wang M., Wu G., Li X., Zhang M., Lu Z., Geng C. (2018). Unraveling submicron-scale mechanical heterogeneity by three-dimensional x-ray microdiffraction. Proc. Natl. Acad. Sci. USA.

[31] Jeong H.T., Park S.D., Ha T.K. (2006). Evolution of shear texture according to shear strain ratio in rolled fcc metal sheets. Met. Mater. Int..

[32] Cabibbo M., Meccia E., Evangelista E. (2003). Tem analysis of a friction stir-welded butt joint of al–si–mg alloys. Mater. Chem. Phys..

[33] Murr L., Liu G., McClure J. (1998). A tem study of precipitation and related microstructures in friction-stir-welded 6061 aluminium. J. Mater. Sci..

[34] Lityńska L., Braun R., Staniek G., Dalle Donne C., Dutkiewicz J. (2003). Tem study of the microstructure evolution in a friction stir-welded alcumgag alloy. Mater. Chem. Phys..

